# Autophagy inactivation in osteosarcoma leads to the appearance of poor prognosis-associated factors

**DOI:** 10.1080/27694127.2022.2112125

**Published:** 2022-09-24

**Authors:** Olivier Camuzard, Marie Nollet, Sabine Santucci-Darmanin, Marie-Charlotte Trojani, Nadia Ben Abderrahman, Virginie Virolle, Fanny Burel-Vandenbos, Sophie Pagnotta, François Orange, Ewa Kolano-Merlin, Georges F. Carle, Valérie Pierrefite-Carle

**Affiliations:** a UMR E-4320 TIRO-MATOs CEA/DRF/Institut Joliot, Université Côte d’Azur, Faculté de Médecine Nice, France; b Service de Chirurgie Réparatrice et de la Main, CHU de Nice, Nice, France; c CNRS, Paris, France; d Service de Rhumatologie, CHU Pasteur, Nice, France; e Plateforme Biologie Moléculaire, iBV, CNRS UMR7277, Inserm U1091, Université Côte d’Azur, Nice, France; f Laboratoire Central d’Anatomie Pathologique, Hôpital Pasteur, CHU Nice, France; g Centre Commun de Microscopie Appliquée, Université Côte d’Azur, Nice, France; h INSERM, Paris, France

**Keywords:** autophagy, osteosarcoma, microenvironment, osteoblast, animal model

## Abstract

Osteosarcoma (OS) is a bone cancer exhibiting a 20% survival rate for metastatic patients, which motivates the development of new therapeutic options. Among the various new treatment approaches, modulation of autophagy is the subject of rising interest. In addition to its pro-survival role in established tumors, autophagy recently emerged as an active player in the crosstalk between tumor and stromal cells. In OS, although the knockdown of key autophagy genes in human cell lines demonstrates a protumoral role of autophagy, the analysis of patient tumors indicates that lack of LC3-positive punctae at resection following neoadjuvant chemotherapy is a poor prognostic marker, suggesting that loss of autophagy is not detrimental for the tumor. In the present work, we analyzed the consequences of autophagy inactivation in OS cells both on tumor development and on bone microenvironment in an orthotopic syngeneic model. We found that inactivation of the autophagy-essential gene *Atg5* in OS cells decreases their tumorigenic properties *in vitro*. However, these effects were no longer observed *in vivo*, likely due to microenvironment modifications such as overexpression of the major OS-promoting factor TGF-β or increased infiltration of Foxp3-positive and CD31-positive cells in *Atg5* KO tumors. In addition, autophagy-deficient tumor cells stimulate the *in vitro* formation of osteoclast, the cells in charge of bone resorption which can release bone matrix-embedded growth factors thereby stimulating tumor growth. Taken together, these results suggest that *Atg5* inactivation in OS cells is associated with microenvironment modifications known as poor prognosis-associated factors in OS, and could thus balance the negative cell-autonomous effects of autophagy suppression.

**Abbreviations**: ACTB -β-actin; Atg -autophagy-related; Baf-A1 - Bafilomycin-A1; CSC -cancer stem cells; Col1A -type 1a collagen; d -day; HBSS -Hank’s balanced salt solution; LC3 -microtubule-associated protein 1 light chain 3 protein; SQSTM1/p62 -sequestosome; OB -osteoblast; OC -osteoclast; OS -osteosarcoma; TEM -transmission electron microscopy; TGF-β -transforming growth factor β; TRAP -acid phosphatase 5, tartrate-resistant.

## Introduction

Osteosarcoma (OS) is a bone cancer, mainly of osteoblastic origin, which primilarly affects children and adolescents [1]. The standard treatment of high-grade localized OS includes multiagent neoadjuvant chemotherapy followed by surgical resection and postoperative chemotherapy, leading to a 5-year survival of 60% [1]. OS patients who poorly respond to chemotherapy exhibit a higher risk of relapse and adverse outcome [2, 3]. Approximatively 15-20% of the patients exhibit a metastatic OS at diagnosis and 25-50% will develop metastasis afterwards, preferentially in the lungs [4]. Survival for patients with metastatic OS or relapse remains low, with an overall 5-year survival rate of about 20% [5], indicating that new therapeutic options are needed [6].

Among the various avenues explored to develop new cancer treatments, autophagy is attracting growing interest [7, 8]. Macroautophagy or autophagy is a major catabolic process used for the degradation and recycling of damaged macromolecules and organelles [9]. This process requires the coordinated action of about thirty autophagy-related (*Atg*) genes allowing the formation of a double-membraned vesicle called autophagosome. The ATG8 protein LC3B (hereafter referred as LC3) is used as an autophagy marker as LC3-II, the lipidated form of LC3 is present on the autophagosome membrane. The cytoplasmic material targeted to degradation is sequestred within the autophagosome which then fuses with lysosome resulting in digestion and recycling of the engulfed material. Autophagy occurs at low level in all cells to ensure the homeostatic turnover of long-lived proteins and organelles, and is upregulated under stressfull conditions for survival purposes [9].

In the context of cancer, most studies have demonstrated that autophagy is used to repress the initial steps in carcinogenesis and/or support the survival and growth of established tumors [10]. In this last case, autophagy was shown to be involved in tumor cell survival, metabolic adaptation, as well as tumor cell motility and invasion [11, 12]. In addition to these established roles, autophagy recently emerged as an active player in the crosstalk between tumor and stromal cells. Indeed, tumor cell autophagy can modify the microenvironment through several mechanisms including secretion, degradation, and signaling modification, leading to modification of immune cell recruitment and activity, and generally resulting in tumor progression [13].

Regarding OS, a study performed on patient tumors demonstrate that a limited number of patient’s pre-treatment specimens exhibited LC3-positive punctae suggestive of relocalization of LC3 on autophagosomes [14]. In addition, lack of LC3-positive punctae at resection following neoadjuvant chemotherapy was an independent poor prognostic marker, suggesting that loss of autophagy could be beneficial for the tumor in OS [14]. Conversely, autophagy was shown to be required for the insulin-like growth factor 2 (IGF2)-induced dormancy-like state which confers OS resistance to chemotherapeutic stress [15]. Moreover, preclinical studies involving the knockdown of key autophagy genes in human OS cell lines demonstrated a protumoral role of autophagy [16, 17]. However, these last studies have generally been performed in nude mice thus eliminating potential immune system effects. In the present work, we analyzed the effect of autophagy deficiency in OS tumor cells in vitro and in vivo, in an orthotopic syngeneic model, as well as its consequences on bone microenvironment.

## Results

### *Atg5* inactivation in OS tumor cells leads to autophagy deficiency

**Figure 1.**
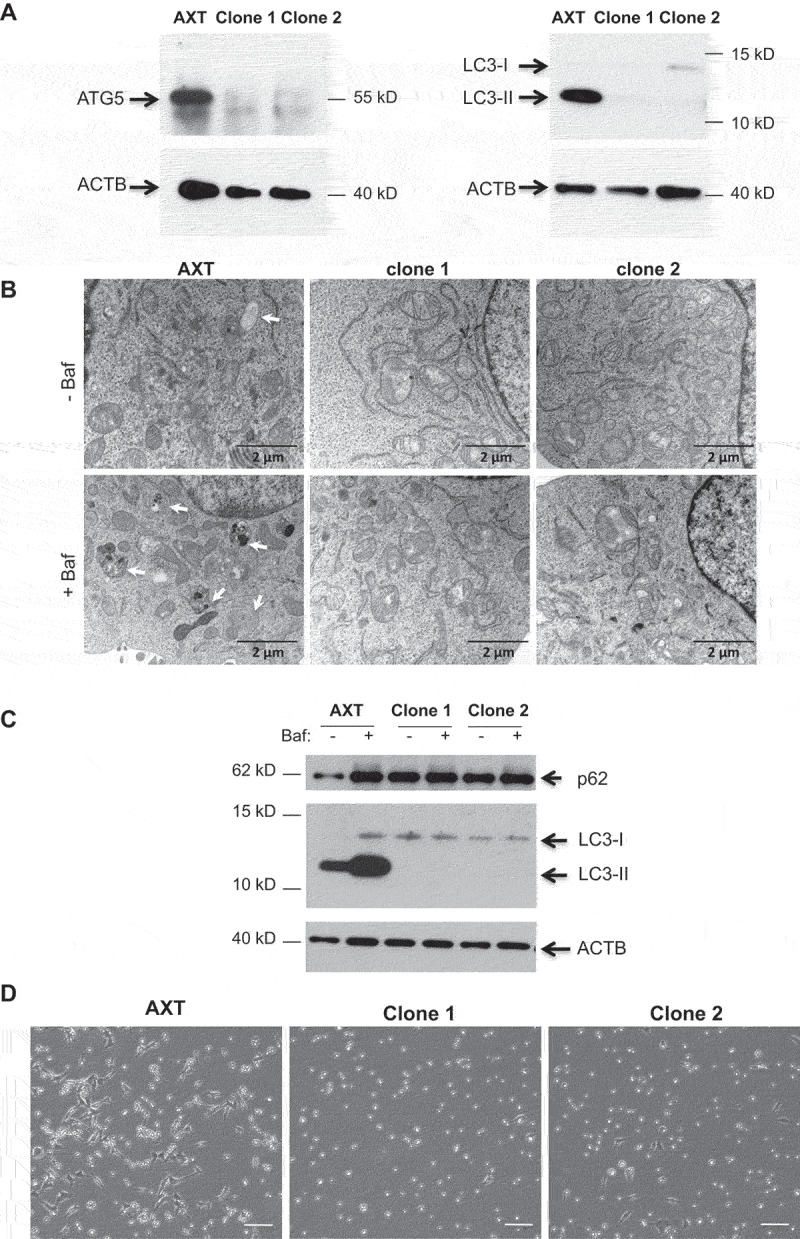
*Atg5* inactivation in OS cells induces autophagy deficiency. (**A**) Western blot analysis of ATG5 and LC3-II in AXT and AXT^Atg5KO^ clones. (**B**) TEM analysis in AXT and AXT^Atg5KO^ clones in the absence or in the presence of Bafilomycin A1 (Baf). White arrow indicates autophagic vesicle. (**C**) Western blot analysis of P62 and LC3-II in AXT and AXT^Atg5KO^ clones after 1h in HBSS in the absence (-) or in the presence (+) of Baf. (**D**) Optical microscopy of WT and *Atg5* KO AXT clones after culture for 24 h in HBSS. White bar corresponds to 100 µm.

To determine the effect of autophagy deficiency in OS, we performed an *Atg5* inactivation in the AXT OS cell line using the CRISPR-Cas9 technology. After transfection of the CRISPR-Cas9 construct and puromycin selection, we screened several clones for the loss of ATG5 by western blot. Two *Atg5* KO clones were then isolated and used in most experiments (clones 1 and 2). As shown Figure 1A, ATG5 expression was lost in both clones and the expression of the autophagosome marker LC3-II was also abolished suggesting autophagy deficiency. To confirm these results, we performed an analysis by transmission electron microscopy (TEM) to visualize autophagic vesicles in the absence or in the presence of Bafilomycin A1, an inhibitor of the lysosomal V-ATPase, which blocks autophagic vesicle maturation. As shown in Figure 1B, Bafilomycin A1 addition in the AXT cells results in the accumulation of autophagic vesicles, which is not the case for the AXT^Atg5KO^ clones. As autophagy can be induced by starvation, we next analysed autophagy in AXT cells and AXT^Atg5KO^ clones after 1h incubation in HBSS in the absence or in the presence of Bafilomycin A1. In the AXT cell line, autophagy was strongly induced as evidenced by the increased LC3-II signal in the presence of Bafilomycin. In contrast, no LC3-II signal was observed in the AXT^Atg5KO^ clones, even in the presence of Bafilomycin, indicating that autophagy is not induced during starvation in these cells. These results are strengthened by the analysis of SQSTM1/p62, an autophagy substrate, which is not degraded in the presence of Bafilomycin A1 in AXT cells, and is accumulated in AXT^Atg5KO^ cells, whatever the condition (Figure 1C).

To analyze the functional consequences of *Atg5* inactivation, we cultured the AXT and the AXT^Atg5KO^ clones for 24 h in HBSS. While some AXT cells are able to resist to this starvation period, this treatment results in the death of AXT^Atg5KO^ cells (Figure 1D).

Taken together, these data indicate that *Atg5* inactivation in the AXT cell line results in autophagy deficiency.

### *Atg5* inactivation in OS cells decreases *in vitro* tumorigenic properties

**Figure 2.**
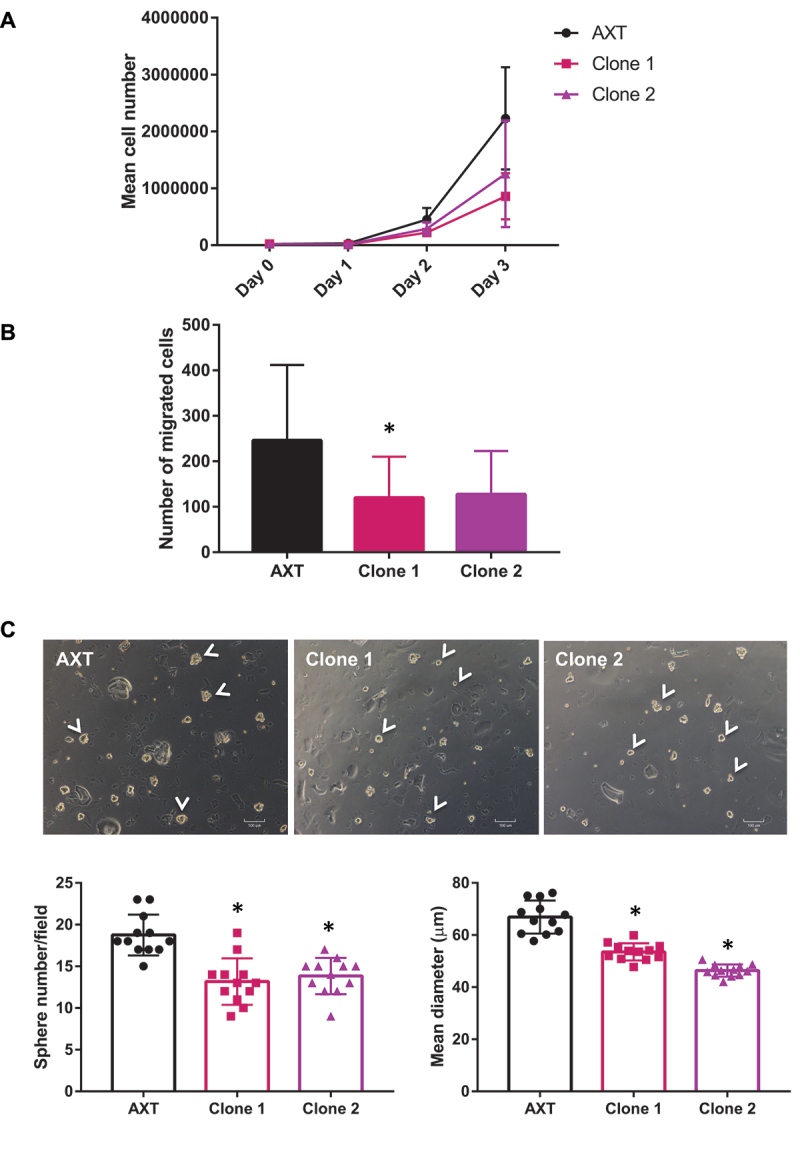
*Atg5* inactivation in OS cells decreases *in vitro* tumorigenic properties. (**A**) Proliferation analysis of AXT and AXT^Atg5KO^ clones for 3 days. Curves represent the mean ± SD of three independent experiments. (**B**) Migration analysis of AXT and AXT^Atg5KO^ clones. Histograms represent the mean ± SD of 3 independent experiments. (**C**) Sphere forming efficiency of AXT and AXT^Atg5KO^ clones. Representative photographs of the spheres (arrowheads) formed after 3 days are presented. Histograms represent the mean ± SD of sphere number and diameter by condition. The number of spheres was counted in 12 different fields by condition. For sphere diameter evaluation, each point represents the mean diameter of 15-18 spheres. *: p<0.05.

To determine the effect of autophagy deficiency on tumorigenic properties, we then compared the in vitro proliferation of WT and *Atg5* KO AXT clones. As shown in Figure 2A, *Atg5* deficiency results in a 40 to 60% decrease of cell number after a 3-day growth period with no increase in mortality, indicating a reduced proliferation. We also compared the migration capacity of WT and *Atg5* KO AXT clones. As shown in Figure 2B, *Atg5* inactivation results in a 50% decrease in the migration efficiency. Finally, we compared the sphere forming efficiency, which reflects the presence of cancer stem cells (CSC) in the cell population. We observed a 25% decrease in sphere number in both AXT^Atg5KO^ clones compared to AXT, and a 20 and 30% decrease in sphere diameter, respectively in clone 1 and 2 compared to AXT (Figure 2C). These results suggest that *Atg5* inactivation in OS cells strongly affects their in vitro tumorigenic properties.

### *Atg5* inactivation in OS cells doesn't impair tumor development and induces TGF-β overexpression

**Figure 3.**
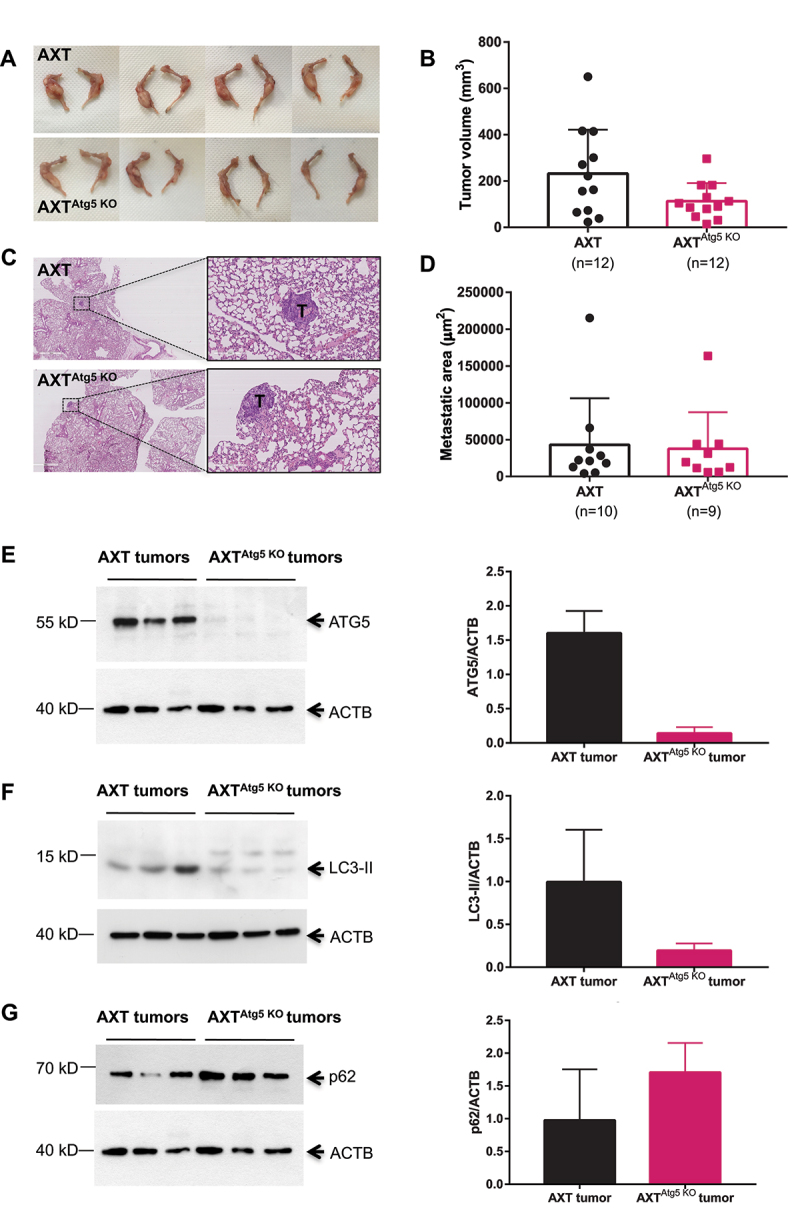
*Atg5* inactivation doesn’t impair OS development *in vivo*. (**A**) Representative pictures of bone tumors obtained after intratibial injection of AXT or AXT^Atg5KO^ cells in syngeneic mice. (**B**) Mean tumor volume in mice injected with AXT or AXT^Atg5KO^ cells. (**C**) Representative pictures of lung metastatic nodules obtained in WT or *Atg5* KO AXT-injected mice. T: tumor. (**D**) Mean metastatic area of lung metastasis in mice injected with AXT and AXT^Atg5KO^ cells. (**E-G**) Western blot analysis of ATG5, LC3-II and P62 in 3 AXT tumors and 3 AXT^Atg5KO^ tumors.

To determine the role of autophagy in OS tumor development in vivo, we then performed an intratibial injection of AXT and clone 1 (AXT^Atg5 KO^) cells in syngeneic mice. Twelve days later, the animals were sacrificed and tumor volumes were measured. Figure 3A shows representative photographs of the tumors, which are composed of an intratibial lesion frequently associated with an external paratibial lesion. No statistically significant difference was observed in the volume of tumors generated by WT or AXT^Atg5 KO^ cells, although there was a tendency to tumor volume decrease in the autophagy-deficient tumor group (Figure 3B). Lung metastasis were observed in all examined animals whatever the group, with 10 lesions in the AXT group and 9 in the AXT^Atg5KO^ group. No difference was observed in the extent of metastatic area in both groups (Figure 3) C and D.

As an alternate explanation for the lack of phenotype *in vivo* could be that the AXT^Atg5KO^ tumors regained ATG5 expression, control and *Atg5* KO tumors were analysed for ATG5, LC3 and p62 expression level. As shown in figure 3, E and F ATG5 and LC3-II expression are almost completely abrogated in the AXT^Atg5KO^ tumors compared to the control ones. Although the results were not statistically significant, we also observed an increase in the p62 expression level in AXT^Atg5KO^ tumors compared to control tumors.

**Figure 4.**
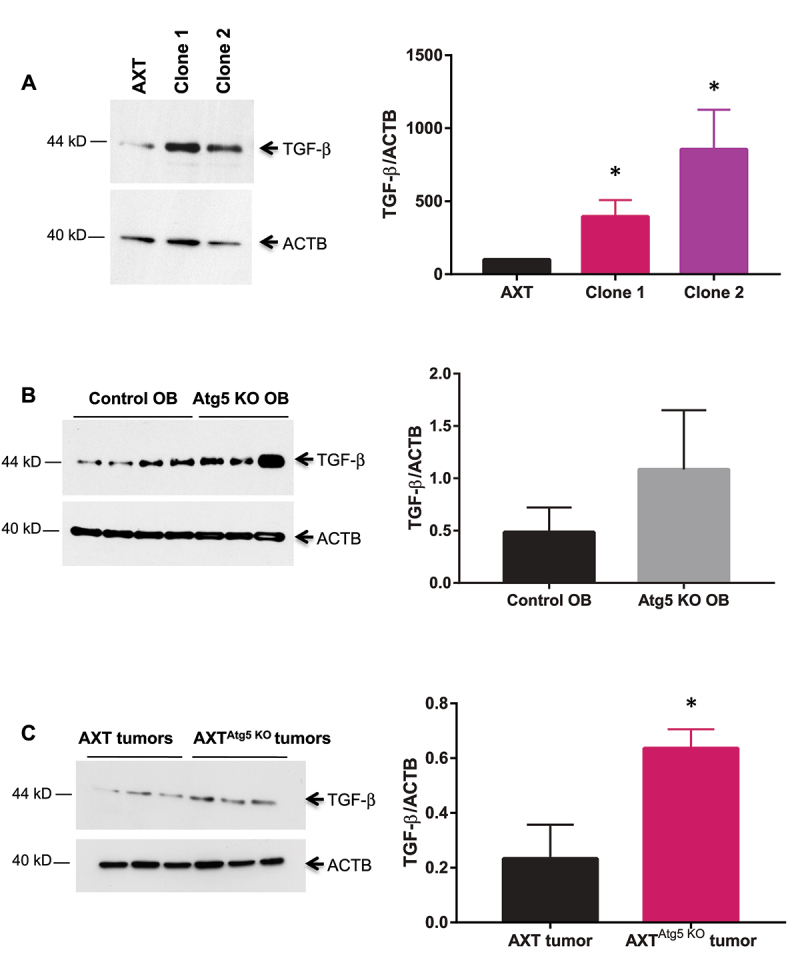
*Atg5* inactivation stimulates TGF-β expression. (**A**) Western blot analysis of TGF-β in WT and *Atg5* KO AXT clones. (**B**) Western blot analysis of TGF-β in WT and *Atg5* KO primary OB. (**C**) Western blot analysis of TGF-β in WT and *Atg5* KO AXT tumors. * p<0.05.

Taken together, these results indicate that autophagy deficiency affects *in vitro* tumorigenic properties but these differences are no longer observed *in vivo*. Thus, we searched for the expression of a protumoral factor overexpressed in AXT^Atg5KO^ cells that could explain these discrepancies. As TGF-β is a major tumor-promoting factor in OS [18]and was recently described as an autophagy substrate [19], we analysed its expression in WT and *Atg5* KO AXT cells. As shown in Figure 4A, we observed a significant increase in TGF-β expression in AXT^Atg5KO^ cells compared to the parental cell line. To determine whether this TGF-β increase was specific to AXT cells or whether it was a more general effect, we analysed TGF-β expression in WT and *Atg5* KO primary osteoblasts (OB) isolated from Atg5*^fl^°^x/fl^°^x^* Col1-Cre- and Atg5*^fl^°^x/fl^°^x^* Col1-Cre+ mice, respectively. We also observed a trend towards an increase in TGF-β in *Atg5* KO primary OB, although the results were not statistically significant (Figure 4B). Finally, we determined TGF-β expression in AXT and AXT*^Atg5^* ^KO^ tumors. As shown in Figure 4C, we also observed an increased TGF-β expression in AXT*^Atg5^* ^KO^ tumors.

### *Atg5* inactivation in OS tumor cells modifies tumor microenvironment

**Figure 5.**
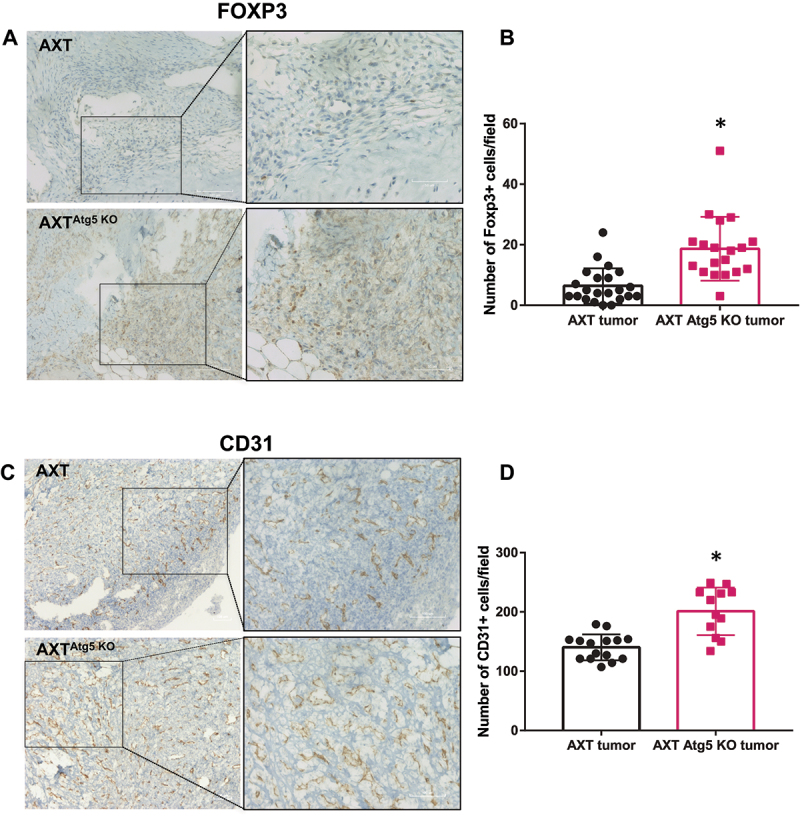
*Atg5* inactivation in OS cells affects tumor microenvironment. (**A**) Representative photographs of Foxp3-positive cells observed in WT and *Atg5* KO tumors. T: tumor. (**B**) Histograms represent the mean ± SD. (**C**) Representative photographs of CD31-positive cells observed in WT and *Atg5* KO tumors. T: tumor. (**D**) Histograms represent the mean ± SD. * p<0.05. n = 3-5 tumors per condition and 3-6 images per tumor.

TGF-β is a key factor in tumor microenvironment regulation through the generation of a systemic immune suppression and the inhibition of host immunosurveillance using various mechanisms [20]. In particular, TGF-β induces Foxp3 expression and generates regulatory T cells (Tregs) [20, 21] which presence in OS microenvironment was associated with a poor outcome [22]. Consequently, we compared the presence of Foxp3-positive cells in WT or *Atg5* KO tumors. As shown in Figure 5 A and B, we observed a significant increase in the number of Foxp3-positive cells in *Atg5* KO tumors compared to WT tumors. As TGF-β is also known to exert pro-angiogenic properties in osteosarcoma [23–25], we next analysed the presence of endothelial cells within the WT or *Atg5* KO tumors. A significant increase of CD31-positive cells was observed in *Atg5* KO tumors compared to WT tumors (Figure 5C-D). These results indicate that autophagy deficiency in cancer cells can promote a tumor-supporting microenvironment.

### *Atg5* inactivation in OS tumor cells modifies bone microenvironment

**Figure 6.**
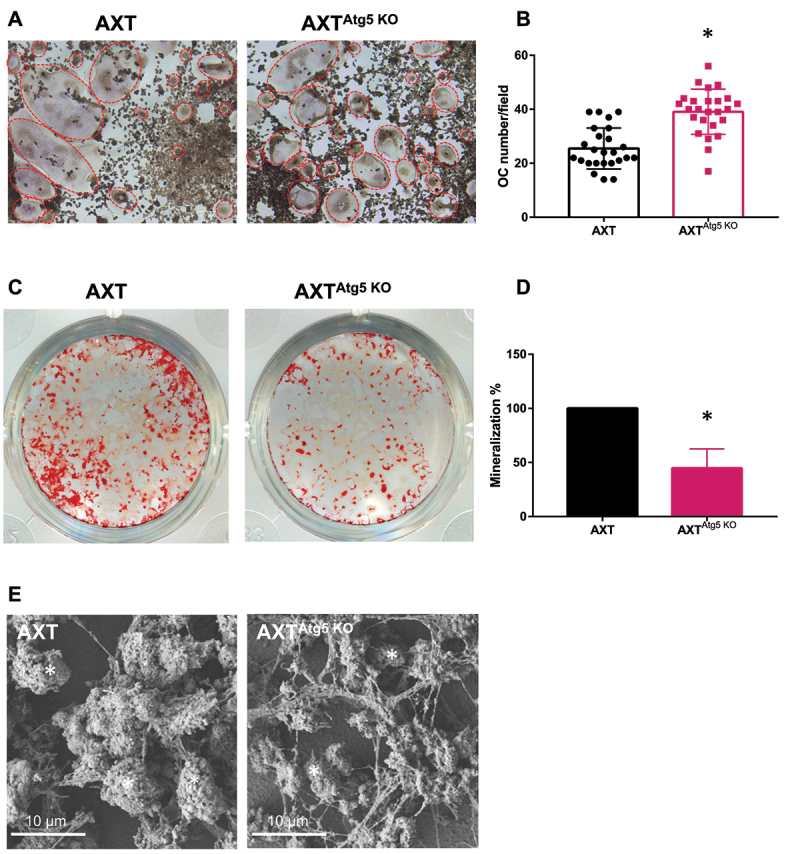
*Atg5* inactivation in OS cells affects bone microenvironment. (**A**) Representative photographs of TRAP-positive cells generated after coculture with WT or mutant OS cells. (**B**) Histograms represent the mean ± SD. Red dashed line: OC identified as multinucleated TRAP-positive cells. * p<0.05. n = 3 wells per condition and 8 images per well. (**C**) Alizarin red S staining of WT and *Atg5* KO AXT cells after 5 days of culture in mineralizing conditions. (**D**) Histograms represent the mean ± SD of three independent experiments. * p<0.05. (**E**) Scanning electron microscopy (SEM) of decellularized matrix generated by WT or mutant OS cells in mineralizing conditions. White asterisk: mineralization nodule.

In OS, the bone tumor microenvironment is also including bone cells, and in particular osteoclasts (OC), the giant multinucleated cells resorbing the bone matrix, as well as a mineralized bone matrix. To determine whether tumoral autophagy can influence bone components of the tumor microenvironment, we next compared the effect of WT or *Atg5* KO tumor cells on OC differentiation in vitro. To this aim, we cocultured WT or *Atg5* KO AXT cells with the RAW 264.7 monocyte/macrophage cell line in the presence of the osteoclastogenic RANKL cytokine. As shown in Figure 6A-B, after a 3-day coculture with *Atg5* KO tumor cells, we observed a 50% increase in the number of tartrate-resistant alkaline phosphatase positive multinucleated cells, suggesting that the presence of *Atg5* KO tumor cells stimulates osteoclastogenesis.

As mineralized matrix is part of the bone microenvironment, we further analyzed the mineralization produced by WT or Atg5 KO AXT cells using Alizarin red S staining. As shown in Figure 6C-D, *Atg5* inactivation results in a 40% decrease in the mineralization capacity. These results were confirmed by scanning electron microscopy analysis of decellularized matrix showing a decrease in the number and the size of mineralization nodules in the mutant matrix (Figure 6E).

## Discussion

Autophagy is known to exert multiple protumoral effects in various types of cancers [10]. In OS, knockdown of key autophagy genes in human tumor cell lines affect in vitro tumorigenic properties [17] and decrease the subcutaneous growth of tumors in immunodeficient mice [16, 17], suggesting that autophagy loss is a disadvantage for these tumors. However, a study of Livingston et al. in 260 human OS tumors, showed that LC3-positive punctae indicative of autophagy were present in only 30% of the pre-treatment biopsy specimens [14]. More importantly, a lack of LC3-positive punctae at resection following neoadjuvant chemotherapy was an independent poor prognosis marker, suggesting that a lack of autophagy could be beneficial for the tumor. In the present work, we analyzed the consequences of autophagy deficiency in OS cells in a mouse orthotopic syngeneic model. We showed that inactivation of the autophagy-essential gene *Atg5* in these cells results in autophagy inhibition as demonstrated by the loss of LC3-II expression in western blot, the absence of autophagic vesicles in TEM, as well as an increased sensitivity to starvation. The *Atg5* KO cells also exhibit a decreased proliferation and migration capacity, which is in agreement with the direct role of autophagy in tumor cell migration through the recycling of components of the cell migration machinery [11, 12]. Indeed, in Ras-transformed breast cancer cells, focal adhesion turnover is mediated by autophagy and is dependent on the autophagy receptor NBR1 [11]. Moreover, autophagy was shown to promote focal adhesion disassembly and cell motility through the direct interaction of Paxillin with LC3 [12]. Finally, we showed that *Atg5* inactivation in OS cells reduces sphere-forming efficiency. Whether the ATG5 KO CSC retain the slower proliferation capacity of their parental cells or whether their reduced development originates from other defects linked to autophagy deficiency remains to be determined. In various cancer types, CSCs were shown to be dependent on autophagy for survival, self-renewal and pluripotency maintenance [26] and we recently showed that autophagy was a crucial process in OS CSC [27]. Taken together, these results indicate that autophagy inhibition in OS cells reduces their in vitro tumorigenic properties. However, no significant difference in tumor development was observed after in vivo OS cell injection, both for the local bone lesion and for lung metastasis. These results suggest that the disadvantage associated with autophagy deficiency could be balanced in vivo by a protumoral factor. One of the candidates was TGF-β, which is produced by OB and regulates the activity of OB and OC to maintain bone homeostasis [28]. Although TGF-β is known to exhibit dual effects in carcinomas, it exerts only protumoral properties in OS [25]. TGF-β levels are increased in the sera of OS patients compared to healthy donors [18]and TGF-β production correlates with tumor aggressiveness and lung metastasis development [18, 29, 30]. Consequently, we compared TGF-β expression level in WT or *Atg5* KO AXT cells and tumors, and we observed a significant increase in the *Atg5* KO samples. In addition, a similar trend towards a TGF-β increase was observed in mouse *Atg5* KO primary OB. Although these results are associative and will require functional studies to validate the proposed model, an increased TGF-β expression after autophagy inhibition had been previously described in vascular smooth muscle cells [31] or in endothelial cells [32] and TGF-β was recently identified as an autophagy substrate in melanoma cells [19]. In the tumor microenvironment, TGF-β plays a critical role, in particular through immunosuppression and angiogenesis promotion [33][20]. Our results indicate that *Atg5* KO tumors exhibit a significantly increased infiltration with Foxp3- and CD31-positive cells. In a multicenter retrospective study, Fritzsching et al. demonstrated that the CD8+/Foxp3+ ratio in OS microenvironment separates survivors from non-survivors, indicating that an increase in Tregs number is associated with a poor outcome [22]. In addition, a high CD31 immunoreactivity was recognized as a significantly worse outcome parameter in OS patients [34]. Hence, autophagy inhibition in OS cancer cells promotes the creation of a tumor-supporting microenvironment. As OS microenvironment is also a bone microenvironment, including OC and bone matrix, we then compared OC formation efficiency in coculture experiments with the RAW264.7 cell line and AXT or AXT^Atg5KO^ cells, in the presence of RANKL. Our results suggest that *Atg5* KO OS cells stimulate osteoclastogenesis. These data could be explained through an increased oxidative stress in these autophagy-deficient cells, as previously demonstrated in mouse *Atg5* KO primary OB [35]. Indeed, reactive oxygen species such as superoxide and hydrogen peroxide promote OC formation and activity [36]. Alternatively, TGF-β, which was shown to exert synergistic effect on OC formation in the presence of RANKL, could be involved in the observed effect [37]. This stimulation of OC generation in the presence of autophagy-deficient tumor cells is of particular interest as a “vicious cycle” has been demonstrated between tumor cells and OC: tumor cells produce cytokines leading to bone resorption and release of bone matrix-embedded growth factors, which in turn promote tumor cell proliferation and further bone destruction [38]. Interestingly, expression profiling of chemotherapy-resistant OS tumors demonstrated that these poor-prognosis lesions are associated with the expression of osteoclastogenesis genes and TGF-β [39].

Finally, we also compared the mineralization efficiency of WT or *Atg5* KO AXT cells and we demonstrated that autophagy deficiency decreases mineralization as previously shown through *FIP200* deletion or *Atg5* inactivation [35, 40].

Taken together, these data indicate that autophagy-inactivation in OS cells modifies the microenvironment therefore supporting tumor development. Although we cannot totally exclude an effect mediated through autophagy-independent ATG5 functions, this hypothesis seems unlikely as ATG5 is involved in activities that could be considered as pro-tumoral such as proliferation or migration/metastasis [41], and its inactivation should therefore inhibit tumor development rather than stimulate it.

In conclusion, autophagy inhibition through *Atg5* inactivation in OS cells impairs tumorigenic properties including proliferation, migration and CSC formation. In vivo, this autophagy defect also modifies the tumor microenvironment by stimulating OC formation and increasing tumor infiltration by immunosuppressive Treg and endothelial cells, potentially through TGF-β overexpression. From a clinical point of view, although this work is performed in a model system using a single cell line with inherent limitations, our results provide new insights on the link between an absence of autophagy and a poor prognosis in OS [14].

## Materials and Methods

### Cell culture

The AXT cell line was isolated after overexpression of *c-Myc* in bone marrow stromal cells from mice exhibiting an homozygous deletion of the *Ink4A/Arf* locus [43,42]. These cells are highly tumorigenic and form lethal OS in syngeneic C57BL/6 mice with pathological features similar to those of human OS. This cell line was maintained in Dulbecco’s modified Eagle medium (Lonza, BE12-604) supplemented with 5% Hyclone fetal calf serum (Thermo Scientific SH30071.03). For mineralization, the cells were cultured in α-MEM (Lonza, BE02-002) supplemented with 5% Hyclone fetal calf serum, CaCl2 (1.4 mM; Merck 2382), ascorbic acid (50 µg/mL; Sigma-Aldrich A4034) and dexamethasone (20 µg/mL; Sigma-Aldrich D8893) for 3 days, and then for 3 additional days in the same medium in the presence of ß-glycerophosphate (50 mg/mL; Sigma-Aldrich G9891). The mouse monocyte/macrophage cell line RAW 264.7 was purchased from American Type Culture Collection and maintained in alpha-MEM supplemented with 5% fetal calf serum.

### Atg5 inactivation using the CRISPR-Cas9 system

Selection of the best sgRNA is designed based on NGG protospacer adjacent motif (PAM) sequences (http://crispor.tefor.net/): 5’-GCCAAGAGTCAGCTATTTGACGTTGG-3’. Amplification of the *Atg5* guide sequence was performed using the following primers [43]: forward 5’-CACCGCCAAGAGTCAGCTATTTGACGT-3’ and reverse 5’-AAACACGTCAAATAGCTGACTCTTGGC-3’. Plasmid used for constructing the CRISPR/Cas9-vector is pSpCas9(BB)-2A-Puro (PX459), a gift from Feng Zhang (Addgene plasmid # 48139; http://n2t.net/addgene:48139; RRID:Addgene_48139). The plasmid is generated by directly ligating synthesized oligos with linearized pX459 vector by BbsI restriction enzyme (Cat: R0539V, New England Biolabs).

Transfection of the CRISPR-Cas9 construct was performed using Amaxa Nucleofector II^TM^ according to manufacturer’s instructions. Briefly, 10^6^ AXT cells were resuspend in 100 µl of V buffer in the presence of 2 µg of the pX459-Atg5 plasmid and submitted to nucleofection (program X-001). A puromycin selection (Invivogene 2.5 µg/ml) was applied for one week, 24-48 h post-transfection and puromycin-resistant clones were selected and amplified before screening by western blot. As the obtained clones exhibit a 50% decrease of ATG5 protein level, one of these clone was re-transfected following the same procedure. The *Atg5*-deficient clones were validated by western blot.

### Proliferation, sphere forming efficiency, and mineralization analysis

Proliferation analysis was performed by seeding 2.10^4^ cells in 12-well plate and the cells were counted every day for 3 days using Scepter^TM^ 2.0 cell counter (Merck Millipore, Molsheim, France) allowing to determine the number of viable cells/ml. Three wells were counted per condition in three independent experiments.

Sphere forming efficiency was determined by culturing WT or *Atg5*-deficient AXT cells (2000 cells/cm^2^) in ultra-low attachment plate (Corning, Fisher Scientific, Illkirch, France) for 3 or 4 days in DMEM-F12 methylcellulose 1%, supplemented with 10 ng/mL bFGF, 20 ng/mL EGF (Peprotech, Neuilly sur Seine, France) and N2 supplement (Invitrogen, Fisher Scientific). Six to twelve fields were analysed by condition at 10x magnification. The number of spheres and the mean sphere diameter was determined in each field.

For mineralization evaluation, the cells were fixed in ethanol 100% for one hour on ice and stained with 1% Alizarin Red S solution (Alfa Aesar, ThermoFisher Scientific) for 10 min. Washes with deionised water were then carried out before the plates were left to dry and photographed. After dissolution in 10% cetylpyridinium bromide overnight à 37°C, the optical density was measured at 557 nm. The obtained results were normalized with DNA content evaluated in unstained wells.

### Obtention of WT or Atg5 KO primary OB and OC generation in the presence of tumor cells

For the obtention of WT or Atg5 KO primary OB, we used *Atg5 ^fl^°^x/fl^°^x^ Col1-Cre+* mice generated by intercrossing the progeny of crosses between *Atg5^fl^°^x/fl^°^x^* mice and α*1(I)collagen-Cre* transgenic mice [35]. Primary OB were isolated from calvariae as previously described [35]. Briefly, calvariae are scrapped with a scalpel, and cut into 1-2 mm^2^ pieces and incubated in alpha-MEM supplemented with 10% fetal calf serum (Hyclone, Thermo Fisher Scientific), glutamine (2mM, Sigma-Aldrich), ascorbic acid (50 μg/mL, Sigma-Aldrich. After 1 week of culture, cells were cultured for 14 days in mineralizing medium consisting of complete medium supplemented with CaCl_2_ (1.4 mM, Sigma-Aldrich) and ß-glycerophosphate (50 mg/mL, Sigma-Aldrich).

For coculture experiments, WT or *Atg5* KO AXT cells were seeded in 24-well plate at a density of 1100 cells/cm^2^. Twenty four hours later, RAW 264.7 cells were added at a density of 30 000 cells/cm^2^ in the presence of GST-RANKL (50 ng/ml) [44]. Three days later, an acid phosphatase 5, tartrate-resistant (TRAP) staining was performed using the leukocyte acid phosphatase kit (Sigma-Aldrich) according to manufacturer’s instructions. Mosaïc images of each well were obtained using Axio Observer Z1 motorized inverted microscope and Zen imaging software (Zeiss). Resulting images were analyzed with ImageJ software (National Institute of Health, USA) to determine OC count in the various experimental conditions. Multinucleated (≥ 3 nuclei) TRAP-positive cells were considered as differentiated OC. OC were counted on 8 images per well and 3 wells per condition.

### Migration experiments

The cells (5.10^4^) were seeded in inserts (8 µm porosity, Corning, Sigma-Aldrich) containing DME without serum. The inserts were then placed in 24-well plate containing DMEM 5% fetal calf serum. After 19h at 37°C, the inserts were rinced in PBS and migrated cells were stained in crystal violet for 15 min. The cells were counted on 3 representative photographs for each insert with 3 inserts per condition in 3 independent experiments.

### Western blot analysis

Cells were washed with phosphate-buffered saline (PBS), scraped in ice-cold PBS and centrifuged at 500 g for 5 min. The cell pellets were resuspended directly in reducing sample buffer (Laemmli: 60 mM Tris-HCl, pH 6.8, 2% sodium dodecyl sulphate (SDS), 100 mM dithiothreitol and 0.01% Bromophenol Blue) in the presence of a complete EDTA-free protease inhibitors cocktail (Roche Diagnostics). Genomic DNA was sheared by passage through a 26-G syringe in order to reduce viscosity. Resulting total protein extracts were then heated at 95°C for 4 min, separated on a 14% SDS-polyacrylamide gel and electrotransferred to polyvinylidene difluoride membranes (Immobilon, Millipore). Blots were blocked for 1 h with Tris-buffered saline-0.05% Tween 20 (TBS-T) supplemented with 5% nonfat milk powder and incubated overnight at 4°C with primary antibodies. Filters were then washed in TBS-T, incubated for 45 min at room temperature with appropriate secondary antibodies conjugated to horseradish peroxydase and washed again prior to detection of signal with ECL plus chemilumiscent detection kit (GE Healthcare). Antibodies used in this study were mouse monoclonal anti-LC3B (M186-3, MBL Clinisciences), rabbit polyclonal anti-ATG5 antibody (Cell Signaling Technology 12994T), rabbit polyclonal anti-p62 antibody (P0067, Sigma-Aldrich), mouse monoclonal anti-ACTB (A1978, Sigma-Aldrich), rabbit monoclonal anti-TGF-β (Abcam ab205604) and goat anti-mouse or goat anti-rabbit horseradish peroxidase-conjugated IgG (Santa Cruz Biotechnology).

### In vivo experiments

The experiments were conducted in accordance with the French and European regulations for the care and use of research animals and were approved by the local experimentation committee (n°2017030710419004). After general anesthesia of C57BL/6J mice, 0.5.10^6^ tumor cells resuspended in 10 µl of PBS were inoculated by intratibial injection in both legs. An analgesia was performed every day by oral administration of buprenorphine (0.4 mg/kg). Twelve days later, mice were sacrificed by cervical dislocation. Tumor volumes were calculated using the formula (l^2^ × L) x 0.5, where l is the smallest and L the largest perpendicular diameter of the tumor. For the analysis of lung metastases, 3 μm-thick sections were generated every 300 μm and the tumor foci were quantified using NDPView2 Hamamatsu software (SZK, Japan).

### Immunohistochemical staining

After a 3 weeks demineralization step in 0,1M EDTA, bone tumors were formalin-fixed and paraffin-embedded blocks were sectioned at 5 μm. Antigen retrieval was performed at 121°C for 15 min in 1 mM EDTA buffer pH 7. Immunohistochemistry was performed using anti-Foxp3 antibody (A54842, Antibodies.com,1:100) or anti-CD31 antibody (281583, Abcam, 1:50), and goat anti-rabbit horseradish peroxidase-conjugated IgG (31460, Thermo Scientific). After staining, the sections were washed in PBS and counterstained with hematoxylin. The positive cells were counted on 3-5 tumors per condition, using 3-9 fields per tumor.

### Transmission electron microscopy

Cells were fixed in 1.6% glutaraldehyde in 0.1M phosphate buffer pH 7.4 immediately after medium removal or centrifugation, respectively. Samples were rinsed with the cacodylate buffer (0.1 M) and then post-fixed in osmium tetroxide (1% in cacodylate buffer) for 1 h. After rinsing with distilled water, they were then dehydrated through an increasing ethanol series and embedded in epoxy resin. Ultrathin sections (70 nm) were collected on Formvar coated copper grids, stained with uranyl acetate and lead citrate and examined with a Jeol JEM 1400 transmission electron microscope equiped with a SIS Morada Camera.

### Scanning electron microscopy

For scanning electron microscopy (SEM), samples were decellularized in 20 mM NH_4_OH, 0.5% Triton X-100 in PBS for 5 min at 37°C, washed in PBS and fixed in a 1.6 % glutaraldehyde solution in 0.1 M sodium phosphate buffer (pH 7.4) at room temperature for 1 hour and then stored at 4°C. After three rinsing in distilled water, samples were dehydrated in a series of ethanol baths (70%, 96 %, 100% three times, 15 min each). After a final bath in hexamethyldisilazane (HMDS, 5 minutes), samples were left to dried overnight. Samples were mounted on SEM stubs with carbon tape and silver paint and coated with platinum (3 nm) prior to observations. SEM observations were performed with a Jeol JSM-6700F SEM at an accelerating voltage of 3 kV.

### Statistical analysis

The results are expressed as mean ± standard deviation. Differences between two groups were compared by the Mann-Whitney test or Student’s t test where appropriate. All statistical analysis were performed with GraphPad Prism (version 7.00, GraphPad Software, Inc., San Diego, CA, USA). P values less than 0.05 were considered significant.
